# Crystallography of honeycomb formation under geometric frustration

**DOI:** 10.1073/pnas.2205043119

**Published:** 2022-11-23

**Authors:** Golnar Gharooni Fard, Daisy Zhang, Francisco López Jiménez, Orit Peleg

**Affiliations:** ^a^Department of Computer Science, University of Colorado, Boulder, CO 80309; ^b^Department of Computer Science, Princeton University, NJ 08544; ^c^Ann and H.J. Smead Department of Aerospace Engineering Sciences, University of Colorado, Boulder, CO 80309; ^d^Department of Physics, University of Colorado, Boulder, CO 80309; ^e^Department of Applied Math, University of Colorado, Boulder, CO 80309; ^f^Department of Ecology and Evolutionary Biology, University of Colorado, Boulder, CO 80309; ^g^BioFrontiers Institute, University of Colorado, Boulder, CO 80309; ^h^Santa Fe Institute, Santa Fe, NM 87501

**Keywords:** honey bees, honeycomb, collective behavior, behavioral assays, mathematical modelling

## Abstract

As honeybees build their nests in preexisting tree cavities, they must deal with the presence of geometric constraints, resulting in nonregular hexagons and topological defects in the comb. In this work, we study how bees adapt to their environment in order to regulate the comb structure. Specifically, we identify the irregularities in honeycomb structure in the presence of various geometric frustrations. We 3D-print experimental frames with a variety of constraints imposed on the imprinted foundations. The combs constructed by the bees show clear evidence of recurring patterns in response to specific geometric frustrations on these starter frames. Furthermore, using an experimental-modeling framework, we demonstrate that these patterns can be successfully modeled and replicated through a simulated annealing process, in which the minimized potential is a variation of the Lennard-Jones potential that considers only first-neighbor interactions according to a Delaunay triangulation. Our simulation results not only confirm the connection between honeycomb structures and other crystal systems such as graphene, but also show that irregularities in the honeycomb structure can be explained as the result of analogous interactions between cells and their immediate surroundings, leading to emergent global order. Additionally, our computational model can be used as a first step to describe specific strategies that bees use to effectively solve geometric mismatches while minimizing cost of comb building.

## Significance

The wax-made comb of honeybees is a masterpiece of animal distributed construction. As bees build their nests in preexisting tree cavities, they grow accustomed to dealing with geometric constraints, resulting in nonregular hexagons and topological defects. We study how bees collectively adapt to their environment by 3D-printing experimental frames with a variety of constraints imposed on the imprinted foundations. The combs constructed by the bees show clear evidence of recurring patterns and can be modeled and replicated through a computational model of crystallographic lattice formation. Our model reproduces global irregularities in the honeycomb structure using local rules and information. It also extends the application of the Lennard-Jones model beyond the physical domain to encompass biological systems, thereby demonstrating its universality.

The wax-made comb of honeybees is constructed distributively by thousands of bees that create a highly regular hexagonal structure (1). This storage structure is essential to the survival of the colony and is constructed in a near-optimal minimization of the wax-to-storage space ratio due to the high energy cost of wax production (2). In particular, honeybees consume about 8.4 lb (3.8 kg) of honey to secrete 1 lb (454 g) of wax (3). This ratio highlights the importance of the geometry of regular honeycomb (4), since a hexagonal tessellation minimizes boundary-per-area (5–7). The regular shape of honeycomb cells has intrigued scientists for centuries, from Darwin, who postulated that colonies with the least amount of honey waste to create the wax comb structure would be most successful (1), to Thompson, who highlighted that honeycombs economize building materials and space (4).

**Fig. 1.**
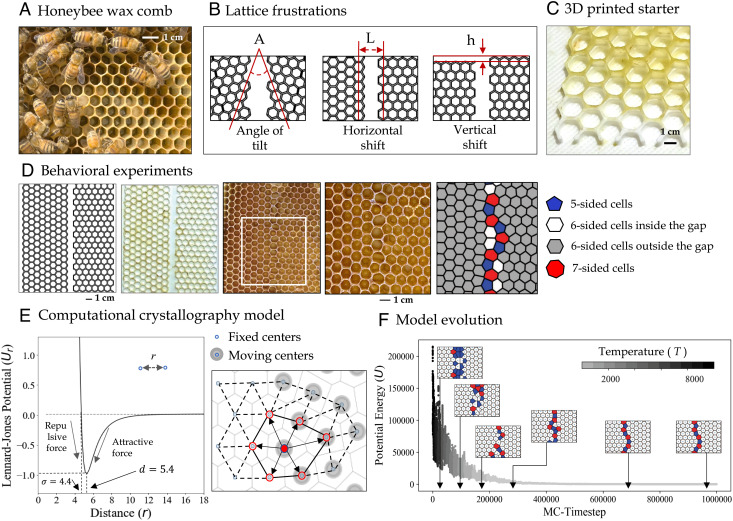
Experiment and model design. (*A*) Collective honeycomb building. (*B*) Illustration of various sources of lattice frustration, namely, angle of misalignment (*A*), horizontal shift (*L*), and vertical shift (*h*), introduced through 3D-printed panels. In all these three scenarios, there is a *gap* in the given foundation that bees fill to achieve a coherent comb structure. (*C*) 3D-printed starter frames thinly coated with beeswax. (*D*) An instance of the behavioral experiments. From Left to Right: a design sheet; the corresponding 3D-printed frame which is coated with a thin layer of wax; the same frame after 25 d when the bees have built comb on it with a white square highlighting the area of interest; the zoomed-in crop of the area of interest; Voronoi reconstruction of the lattice [showing defects in red (*Z* >  6) and blue (*Z* <  6), where the topological charge *Z* is equal to the number of cell sides]. (*E*) Description of our computational model that works according to the rules of the Lennard-Jones potential model. On the left is the graph of the Lennard-Jones potential function: potential energy *U*_*r*_ as a function of the distance (*r*) of a pair of particles describes both the attractive and repulsive forces between particles. The box on the right shows an illustration of a section of the model with moving and fixed centers highlighted in gray and black. The position of the moving center shown in solid red can be changed at each time step by calculating the potential of its immediate Voronoi neighbors, highlighted with arrows and red rings around them. (*F*) As the model evolves and the system cools within a simulated annealing run, the potential energy decreases. The inset pictures illustrate gradual convergence of the comb structure as the model runs through the optimization process toward the minimum energy state.

Perhaps even more enigmatic than the regular structure of the comb is the distributed nature of its construction, where worker bees—akin to distributed wax 3D printers—simultaneously manipulate small pieces of wax to collectively construct a coherent comb structure (Fig. 1*A*). As honeybees are able to build their nests in preexisting tree cavities, they deal with situations that do not allow for a regular hexagonal lattice, such as the presence of boundaries or structures that require them to combine cells of different shapes and sizes, which results in nonregular hexagons and topological defects (8, 9). Several observations of the structure of the comb (10, 11) suggest that modifications to the regular pattern extend over several cells. This reinforces the hypothesis that a long-range awareness of possible constraints results in an adaptive collective behavior with the goal of minimizing the use of wax. While there have been extensive studies focused on the geometry of the hexagonal regular unit cell (12–15), the mechanisms controlling the density and distribution of defects in the lattice are still not well understood. Only in the last decade have there been studies characterizing irregular patterns quantitatively (9, 11, 16, 17), and the rules governing the planning and construction of honeycomb under external constraints incompatible with a regular lattice remain an open question (18, 19).

In this work, we leverage 3D printing and rapid prototyping to design repeatable experiments with precisely controlled and carefully quantified sources of geometric frustrations, illustrated in Fig. 1*B* and *C*. This approach makes it possible to systematically vary a single parameter between different experiments and study its effect on the resulting honeycomb structure. Furthermore, the repeatability enabled by these 3D-printed frames allows us to study statistical variations among experiments with identical initial conditions. We process the resulting patterns using quantitative tools that, while used extensively in crystallography to study lattices in inorganic systems, are not commonly applied to the structures found in animal architecture. In particular, we focus on characterizing the topological defects (i.e., cells with more or less than six neighbors) that appear as a consequence of the imposed sources of frustration, such as those in Fig. 1*D*.

On the modeling front, we also make use of tools from the field of crystallography. We use an algorithm based on simulated annealing to optimize the position of cell centers so that they minimize a variation of the Lennard-Jones potential, which has previously been used to model similar crystallographic structures in graphene (20). Recent efforts to explain irregularities in honeycomb structure optimized the geometry within a fixed initial topology of the cell lattice (11), which could effectively restrict the configuration space. In contrast, our model optimizes the geometry and topology of the lattices simultaneously by allowing the interconnections between the cells to change and adapt to the nearest neighbors’ potential. It is worth noting that the presented model does not attempt to explain the rules of interactions between bees or their decision-making process. Instead, the bees are implicit in it, and the model focuses on describing the position, shape, and size of the cells of the honeycomb after it is built under each controlled source of geometric frustration. Conceptually, the implicit bees would effectively position the cells according to the local interactions with adjacent cells, rather than explicitly build the cell walls (as defined in *Methods*). Therefore, identifying (through experiments) and reproducing (with the model) the density and distribution of defects in the structure of the honeycomb can provide an explanation regarding the underlying mechanisms behind the efficacy and effectiveness of the structure of the honeycomb even when it is built in the presence of specific geometric frustrations.

## Methods

We take a perturbation approach to reverse-engineer the local rules that lead to honeycomb construction: by perturbing the system with carefully prescribed conditions, we anticipate gaining a deeper understanding of the underlying principles of distributive construction. In other words, we aim to study how bees overcome scenarios in which engineered constraints make it impossible to build a regular hexagonal lattice. Our focus is on three different cases of frustration, namely angle of tilt (*A*), horizontal shift (*L*), and vertical shift (*h*) (illustrated on sections from our design sheets presented in Fig. 1*B*). Both the behavioral experiments and our computational model are designed in a way that enables us to independently vary the values of these three parameters and study their impact on the honeycomb lattice.

### Behavioral Experiments

We introduce geometric frustration to the system on a microscopic scale (i.e., on the scale of individual cells) using 3D-printed foundation plates. The printed foundation is only introduced to segments of the plate, which are separated by gaps with no pattern. The geometry and patterning of the panels are deliberately designed so that the bees will not be able to simply extend the provided hexagonal foundations to fill the gaps and connect the patterned regions. Fig. 1*D* shows an instance of a behavioral experiment, starting from a design sheet followed by the corresponding 3D-printed plate, which is provided to the bees as a starter frame. We then take pictures of the fully built frames and identify areas of interest in each image. We define this area as the largest crop on each plate that contains an undamaged comb with cells within and on either side of the gaps, highlighted with a white rectangle in the middle image in Fig. 1*D*. We use computer vision techniques to automatically identify individual bee comb cells on the selected crop. For details and examples of the steps in this process, please refer to *SI Appendix*, Fig. S2. The final image in the series in Fig. 1*D* is an example of the output of the image processing procedure: a Voronoi tiling based on the cell centers, where each comb cell is replaced by the corresponding Voronoi cell which reveals the nonregularity of the shapes of cells within and around gaps. We find striking agreement between the Voronoi construction and the network of honeycomb cells built on the experimental frames under various conditions. Nevertheless, to confirm that the Voronoi reconstruction matches the actual comb image, we visually inspect each image with the Voronoi diagram overlaid and make corrections if necessary. Once the corrected Voronoi tessellation is created, it can be used to calculate geometric and topological properties of the cells such as coordinates of the cell center, topological charge *Z* (i.e., number of cell neighbors), and cell area.

### Computational Crystallography Model

Our goal in this section is to develop a physics-based mathematical description of a set of rules governing honeycomb construction at the local scale that can explain the global patterns we observe in our experiments. To that end, we establish a computational model solved via simulated annealing, a technique for approximating the global optimum of a function (21, 22) that is based on Monte Carlo methods and was originally developed to generate sample states of a thermodynamic system (23). It receives its name from the similarity to the process of annealing in materials science and is often used to study the formation of crystals resulting from the minimization of a potential energy (24–26) or to reconstruct the microstructure of dispersions and heterogeneous solids (27–29). It requires defining a function to be minimized (analogous to the internal energy in a crystal), which depends on the state of the system. In our case, state variables are the position of the centers of honeycomb cells, which are used to define a potential energy function *U*. The method minimizes the potential by exploring neighbors of the current state of the problem, in which particles are disturbed by a small displacement. Each displacement is accepted according to a probability *P* given by:


[1] $$\begin{array}{c} P=\exp\left(-,\frac{\Delta U}{T}\right), \end{array}$$

where *ΔU* is the change in potential energy due to the change from the current to the new proposed state, and *T* is a global parameter that controls the probability of accepting changes in state that increase the energy, and is analogous to temperature in real physical material annealing. Temperature is traditionally decreased during the process. It is initially high, so that the initial exploration of the solution space accepts a wide range of possible states, including some that increase the energy. The temperature then decreases as the solution converges to an optimum, so that only changes that minimize the energy are accepted, as in Fig. 1*F*. The specific range of values depends on the problem and is often unrelated to realistic temperature values in metallurgy processes.

The minimized potential is a variation of the Lennard-Jones potential (Fig. 1*E*), known to produce hexagonal lattices in the absence of constraints. The interaction between particles is given by:


[2] $$\begin{array}{c} U\left(r\right)={\left(\frac{\sigma }{r}\right)}^{12}-{\left(\frac{\sigma }{r}\right)}^{6}, \end{array}$$

where *r* is the distance between two particles that are identified as first neighbors by a Delaunay triangulation (i.e., we ignore long-range interactions), and *σ* is the distance at which the potential is zero, *σ* = *d*/(2^1/6^). The potential is minimized at a particle-to-particle distance *d* = 5.4 mm. We define this as the distance between the center of the cells built by bees under no geometric frustration, which is measured directly in the regular honeycomb produced in our control experiments. We assume this distance to be constant throughout our simulations, regardless of the imposed frustration. The Delaunay triangulation is updated at each step, to account for the interplay between geometry and topology in the network as the distance between particles changes. The other main parameter in the Lennard-Jones model is the dispersion energy or depth of the potential well, usually represented as *ϵ*. Since this value results in a constant scaling of the energy values, it does not fundamentally alter the energy landscape or the position of particles leading to local minima at the end of the optimization process. As such, it has no effect on the results of this model, so it does not appear in Eq. 2.

The model simulates the same constraints used in the experiments: misalignment angles, *A*, and horizontal and vertical shift of the hexagonal lattices, *L*, *h*. The algorithm considers two types of particles: the center of the cells imprinted in the starter panel, which act as a set boundary (i.e., fixed centers, shown with a blue dot in Fig. 1*E*), and the center of the cells that are created in the gaps, which are the variables in the minimization process (i.e., moving centers, highlighted with a gray shade around them in Fig. 1*E*). Two edges of the simulation box are bounded by fixed particles, corresponding to the cells provided in the printed frames. The cells that are free to move are repealed by those cells due to the potential energy. We assume nonreflecting walls on the other two boundaries, which means that we simply limit the movement of the cell centers beyond the edges of the simulation box. The initial arrangements of the fixed cells in the model are chosen to replicate the scenarios explored with our experiments. The number of moving centers in the simulations depends on the size of the system, as well as the specific type of geometric frustration being explored, and is based on the number of cells created by the bees in our experiments (see *SI Appendix*, Fig. S9 for a complete list of these values derived from the experiments for each parameter combination). At the end of each simulation, the arrangement of the cells reaches an equilibrium configuration that can be directly compared against the configurations bees create in the corresponding experiments.

## Results

**Fig. 2.**
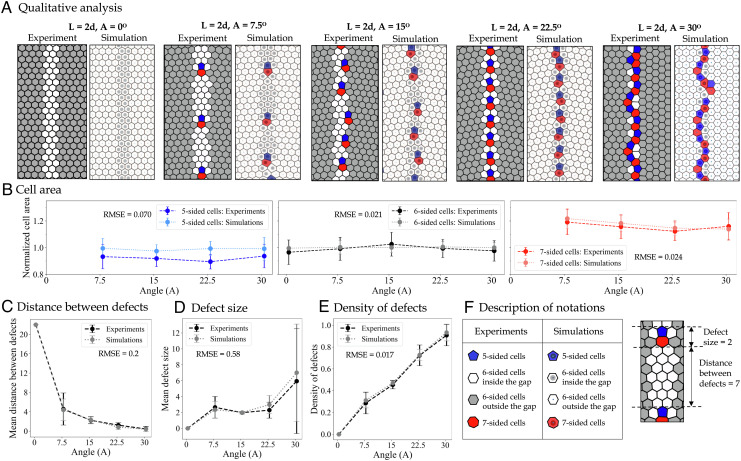
Results for varying the misalignment angles (0 ° ≤*A* ≤ 30°). (*A*) Pairs of images taken from experiments (on the left) and simulations (on the right) to highlight the qualitative agreement between our model and experiments. Defects are nonhexagonal cells, shown in red (*Z* >  6) and blue (*Z* <  6), where *Z* is the topological charge (i.e., number of sides) of each cell. (*B*) Mean cell area across different angles categorized by the cell shape, shown both in experiments (dashed lines with darker colors) and model (dotted lines and brighter colors). (*C*) Mean distance between defects in experiments (dashed black) and simulations (dotted gray) shows a decline as the angle of misalignment increases. (*D*) Average length of defect chains in experiments (dashed black) and simulations (dotted gray) generally increases as the angle of misalignment increases. The large error bars in the case of *A* = 30 suggest the presence of defective chains of various sizes when the angle of misalignment is large. (*E*) Density of defects (i.e., the number of nonhexagonal cells divided by the height of the crop) shows a sharp increase as the angle of misalignment increases. To quantify the agreement between experiments and model results, the value of root mean squared error (RMSE) is calculated and shown for all the parameters plotted in panels *B*–*E*. The small values of RMSE indicate that our model can predict the experimental data relatively accurately. (*F*) The table on the left shows a description of the notations used in panel *A* to distinguish between various cell types. On the right is an example of how the two variables in panels *C* and *D* are calculated.

**Fig. 3.**
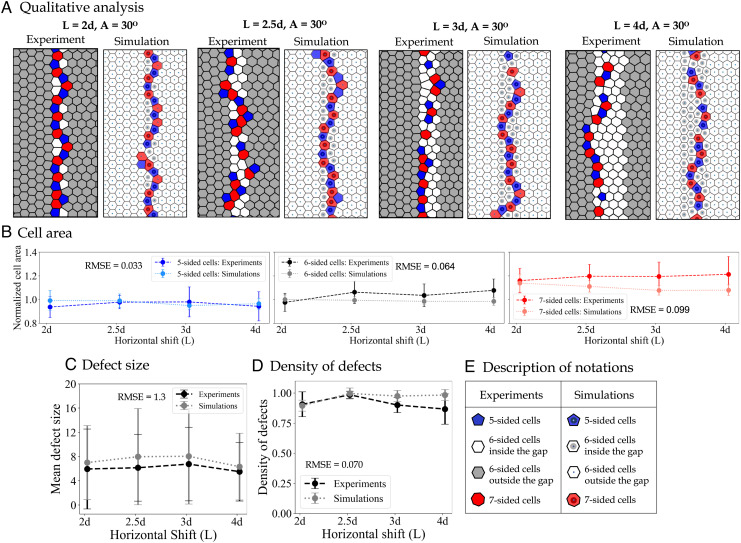
Results for varying the horizontal shift parameter 2*d* ≤ *L* ≤ 4*d*. (*A*) Pairs of images taken from experiments (on the left) and simulations (on the right) to highlight the qualitative agreement between our model and experimental results. Defects are shown in red (*Z* >  6) and blue (*Z* <  6), where *Z* is the topological charge (i.e., number of sides) of each cell. (*B*) Mean cell area across different values of vertical shift categorized by the cell shape, shown both for experiments (dashed lines with darker colors) and model (dotted lines and brighter colors). To see the distribution of cell sizes, refer to *SI Appendix*, Fig. S5. (*C*) Average length of defect chains in experiments (dashed black) and simulations (dotted gray) shows small changes for various horizontal distances between panels. The large error bars indicate the presence of defective chains of various sizes when the angle of misalignment is *A* = 30. (*D*) Density of defects (i.e., the number of nonhexagonal cells divided by the height of the crop) shows a high density of defective cells within the gap. The density of defects stays relatively high as the value of the horizontal shift increases. (*E*) Description of the notations used in panel *A*.

**Fig. 4.**
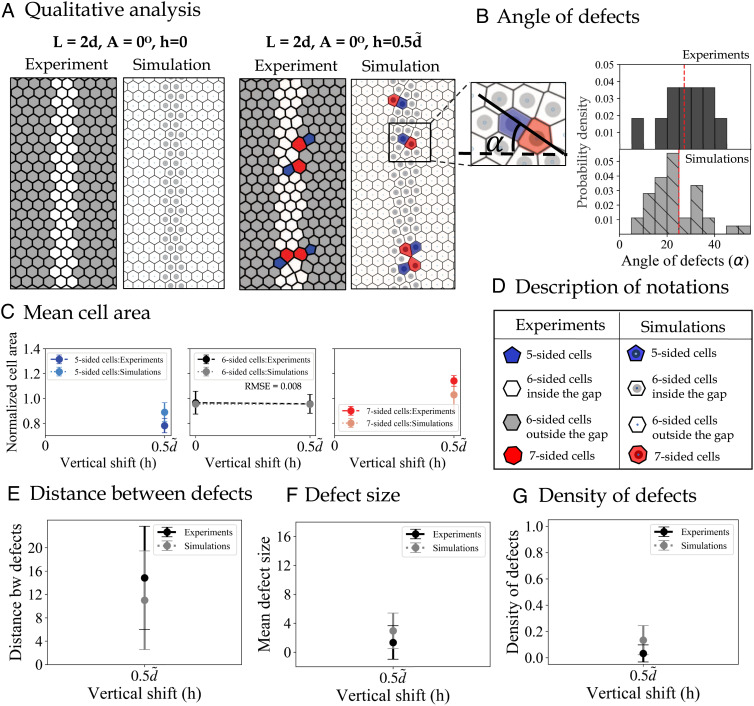
Results showing the impact of vertical shift *h* = 0, 0.5*d*. (*A*) Pairs of images taken from experiments (on the left) and simulations (on the right) which highlight the qualitative agreement between our model and experimental results [showing defects in red (*Z* >  6) and blue (*Z* <  6), where *Z* is the number of cell sides]. (*B*) Probability density of the angle of defects *α* shows very similar mean and SD values across both experiments and simulations. *α* = 24.97 ° ±10.03° in the experiments and *α* = 27.14 ° ±11.05° in simulations, and 0 ° < *α* <  90°. The mean values are highlighted with dashed red lines on both plots. (*C*) Mean cell area categorized by the cell type, shown in experiments and model for the two values of *h*. (*D*) Description of the notations used for various cell types shown in panel *A*. (*E*) Mean distance between defects in experiments (dashed black) and simulations (dotted gray). (*F*) Average size of connected defective cells in experiments (dashed black) matches with our simulations (dotted gray) (*G*) Density of defects (i.e., the number of nonhexagonal cells divided by the height of the crop).

In this section, we present our experimental and modeling results, focusing on the effect of varying each of the sources of geometric frustration, namely misalignment angles (*A*), horizontal shift (*L*), and vertical shift (*h*) of the regular lattices. The horizontal shift is expressed as a function of *d*, the distance between the centers of two adjacent hexagons in the regular lattice, which is used to define our simulation framework, as described in the previous section. The vertical shift will be expressed as a function of $\tilde{d}=\sqrt{3}\;d/2$, the vertical distance between two adjacent cells at 30° from each other. The results that follow use data collected from a total of 10 *Apis mellifera* honeybee hives that built comb on the 3D-printed experimental frames with varying values for the three parameters described above. The crops that show the qualitative agreement between the model and experiments are presented in panel *A* of Figs. 2–4. These results display the most common patterns that we observed in both simulations and experimental data under each condition. A quantitative analysis of these results is performed on a larger dataset containing all the experimental data (3–5 crops of the same size for each parameter combination) and 10 simulation runs. The largest common crop size across all the experimental data displayed in this work contains 22 rows of cells. To generate model results, the size of the simulation box is set to match the experimental crops for better visual comparison. All plots show the average of all identical experiments and simulations, with the SD as error bars. Finally, in order to verify that the resulting patterns are not affected by external factors (e.g., material used in 3D printing) and arise from the geometric frustrations that were imposed on the starter frames, we 3D-print several control frames without any geometric frustrations (see *SI Appendix*, Fig. S1 for instances of experimental and control frames) and place them in all of the hives along with the experimental frames. Unsurprisingly, we find that the bees consistently build regular and perfect comb on the control frames without any defects. Please see *SI Appendix*, Fig. S3 for the results of running the image processing pipeline and automatic cell detection on an instance of a control frame.

### Exploring the Impact of the Angle of Misalignment (A)

Fig. 2*A* shows the qualitative comparison between the processed experimental frames and the simulation output with various values of tilt. In order to focus on the impact of specific angles in the pattern of the cells that connect the gap between the panels, Fig. 2*A* reports instances of both experimental and model results with varying angle (0 ° ≤*A* ≤ 30°) but with a fixed distance of *L* = 2*d* between the panels. We show five pairs of images in Fig. 2*A*, with Voronoi reconstruction of the experimental data on the left. The shades of gray in the experimental reconstructions represent the cells built on the given 3D-printed foundations, whereas the regular cells built inside the gap with no foundation are shown as white. In the model results, shown on the right-hand side of the pairs in Fig. 2*A*, the moving lattice elements (equivalent to cells built in the gap) can be distinguished by their darker centers. Both the model and the experiments resolve the frustration through the introduction of topological defects (i.e., cells with other than six neighbors). These often take the form of dislocations, i.e., a duplet of a positive and a negative (5–7) defect.

The results in Fig. 2*A* show that increasing the misalignment angle increases the linear density of defects (i.e., decreases the distance between pairs) with excellent quantitative agreement between the model and the experiments. We also explore the variation in cell sizes by calculating cell areas across all angles and categorizing the results based on the number of sides, as shown in Fig. 2*B*. Unsurprisingly, cells with a greater number of sides (shown in red) are on average larger. This pattern persists across different angles of tilt, both in experiments and simulations. However, the distribution of cell sizes shown in *SI Appendix*, Fig. S4 denotes that there is more variability of the cell sizes in the collected experimental data compared to the cell sizes generated in the model. This can be due to specific functionality of cells of various sizes and shapes within the hive. For instance, we find that many of the larger hexagons and heptagons that emerge as a result of tilted patterns on the experimental frames are used as drone comb (see *SI Appendix*, Fig. S8), which typically consist of larger cells for the queen to lay unfertilized eggs in. Fig. 2*C* captures the opposite correlation between increasing the angle (*A*) and the distance between the defects. The distance between defects is calculated by counting the topological distance between two sets of defective cells; see Fig. 2*F* for an example of a distance of seven cells between two dislocations. Furthermore, we plot the change in the average size of defect chains as a function of the angle of tilt in Fig. 2*D*, which shows very small variation for small angles, when we observe mostly dislocations, and a positive correlation for large angles, when we observe large defect chains. The size of a defect chain is calculated by counting the number of connected (uninterrupted) nonhexagonal cells. See Fig. 2*F* for an example of a defect size of 2. Fig. 2*E* highlights the positive correlation between the number of defective cells per crop and the angle of tilt. Since there are no defects in the case of *A* = 0°, this angle is not included in the statistical analysis of the defects shown in Fig. 2 *C*–*E*.

### Exploring the Impact of Horizontal Shift (L)

Fig. 3*A* illustrates how the structure of the comb is impacted by keeping a fixed angle of tilt *A* = 30° across all instances while increasing the distance between them in the range 2*d* ≤ *L* ≤ 4*d*. We also performed experiments in which the distance between the given hexagonal lattices went up to 11*d*. However, during the time frame of our experiments, the bees did not connect the two lattices if the distance between them goes beyond *L* = 4*d*. See *SI Appendix*, Fig. S7 for examples of such incomplete trials with large gaps between the given foundations. In fact, even within the range of 2*d* ≤ *L* ≤ 4*d*, the size of our experimental dataset shrinks as we increase the distance, with fewer analyzable samples for *L* = 4*d*. As Fig. 3*A* illustrates, bees build long chains of alternating 5–7-sided cells inside the gaps across various distances when the angle of tilt is large (*A* = 30 ° ). When the distance between the panels is larger than *L* = 2*d*, the bees continue to build hexagons even when there is no foundation underneath and then use a chain of 5-7-sided cells to connect the hexagonal lattices on either side of the gap.

Comparison of cell sizes, shown in Fig. 3*B*, confirms the positive correlation between the number of sides and cell area, which is quantified in the previous section as well. This pattern holds the same across various distances, both in the experiments and in the model results. Fig. 3*C* indicates that the average defect size does not change substantially across varying distances. This is mainly because the angle of tilt is constant (*A* = 30°), so the bees continue to build their preferred shape (hexagons) within the gap (regardless of the horizontal distance between the panels) and finally use the long chains of defective cells to combine the two sections of the comb. The relatively high but constant density of defects, shown in Fig. 3*D*, confirms the qualitative results shown in panel *A*, suggesting that horizontal shift between panels does not impact the size or the density of defects as long as the angle of misalignment is fixed. In fact, our experimental data (*SI Appendix*, Fig. S6) confirms that bees build the same patterns of defective cells, shown in Fig. 2*A*, to combine the two lattices of various angles of misalignment even when the distance between the two lattices is larger than *L* = 2*d*. However, there could be some significance to the position of the irregular cells built in the defective chains both in the model and experiments (see *SI Appendix*, Fig. S10 for more details). A careful analysis of this would be an important future direction of our work.

### Exploring the Impact of Vertical Shift (h)

In this section, we explore the impact of imposing a vertical shift, *h*, between the panels on either side of the gaps. To highlight the isolated effect of *h*, we illustrate two conditions, namely *h* = 0, where there is no vertical displacement of the panels, and $h=0.5\tilde{d}$, while assuming a fixed value of *A* = 0°, and *L* = 2*d*, so that the vertical shift is the only source of frustration. Our qualitative results for these two scenarios are shown in Fig. 4*A*, which demonstrates great agreement between experiments and simulations showing more defective cells used for filling the gap between the given hexagonal regions when there is a vertical shift in the structure of the panels. As shown before, there are no defects built in the gap when *L* = 2*d*, *A* = 0°, and *h* = 0. However, the pairs (or short chains) of defective cells are built inside the gap as a result of a nonzero value of *h*. We also notice the common tilt of these often short chains of defects and quantify it across both experiments and simulations. Fig. 4*B* shows two distributions for the angle of defects across experiments (solid bars) and simulations (hatched bars) with a similar mean value highlighted with a dashed red line on both plots (i.e., *α* = 24.97° in the experiments and *α* = 27.14° in simulations). The average cell size plots shown in Fig. 4*C* follow the same trend as described before, where the cells with fewer sides are on average smaller. Since there are no defects in case of *A* = 0, *h* = 0, only six-sided cells are shown in these size plots. The average distance between defects, defect size, and density for *A* = 0, *h* = 0.5*d* are shown in Fig. 4*E*–*G* for experiments and simulations. The large value of the average distance between defects shown in Fig. 4*E* suggests sporadic, small chains of defects when *h* = 0.5*d*, which is confirmed in Fig. 4*F* and *G*. Interestingly, even if the distortion created by vertical shift could be resolved with deformation of the hexagonal cells without introducing topological defects, we observe similar nonzero defect density in both experiments and simulations. It is not clear whether the presence of defects is used to reduce distortion of the hexagonal cells or to create a variation in cell size for different functionality.

## Discussion

Several processes in nature result in the formation of self-organized lattice patterns, including graphene at the nanoscale (30, 31), colloidal crystals at the microscale (32, 33), and elastic dimples in soft bilayers at the macroscale (34, 35). In the absence of external constraints, the resulting pattern is often a topologically and geometrically regular crystal lattice (36). However, topological defects can appear due to different sources of geometric frustration, such as incompatibilities between two crystalline regions with different orientations (37–43).

In this work, we observed similar defect formation in the honeycomb structure. We have used 3D-printed panels with precisely engineered constraints to confirm that bees consistently build specific patterns of topologically irregular cells to solve various geometric frustrations, which can then be quantified through crystallography tools. Inspired by the similarities between the grain boundaries in our system and those observed in graphene (44), we have used a variation of the Lennard-Jones potential to model honeycomb formation under geometric frustrations. The agreement between experiments and predictions highlights the potential of using tools from crystallography to rationalize the comb construction process. In particular, our results demonstrate that the apparent long-range order observed in the honeybee comb can be explained as the result of local rules. The similarities between the defect patterns in honeycomb and other systems further reinforce previous observations that defect formation is relatively insensitive to the type of interaction between lattice elements (45, 46).

While our work was in progress, we became aware of a related study by Smith et al. (11). Our conclusions are in agreement with respect to the consistent patterns of 5- and 7-sided cells that appear to accommodate misalignment between regular regions, which in their study was observed in the absence of a frame foundation. In addition, we have shown that using 3D-printed foundations to introduce engineered and repeatable geometrical frustration makes it possible to study statistical variations among experiments and to directly compare with modeling predictions. Lastly, our model optimizes the geometry and topology of the lattices simultaneously by updating the connectivity between the cells as their geometry evolves during the annealing, which avoids the risk of overconstraining the pattern due to a fixed topology.

The structure of the honeycomb is the result of complex, effective interactions between honeybees as well as with the existing cells and their environment. A detailed description of the bees’ behavioral rules that leads to collective comb construction is challenging to capture experimentally. Therefore, we simplify the modeling framework by considering *implicit bees* that position the cell’s centers according to the local physical interactions, rather than considering how bees deploy wax to construct the *explicit walls* of the hexagonal lattice. In the future, our experimental-modeling framework could be extended by making use of potentials directly addressing bee behavior (e.g., minimizing wax use or variations in size) to better understand various aspects of the construction process that can affect the global honeycomb patterns. These include the relationship between the cells and their location (e.g., do bees build differently on the top of the frame vs. the bottom of the frame?), the effect of the sequential nature of cell construction (e.g., do bees fix already built cells when an obstacle is found?), and the fluctuating colony requirements for specific cell functionality (e.g., do bees build differently when the colony needs to store more honey?). Overall, our interwoven experimental-modeling framework paves the path toward exploring the rich and complex aspects of the honeycomb formation process.

## Supplementary Material

Appendix 01 (PDF)

## Data Availability

The data (47) and code used in this study for image analysis and automatic cell detection on 3D-printed frames, as well as the code for our computational model, have been deposited in GitHub at https://github.com/peleg-lab/Honeycomb.git.

## References

[1] C. Darwin, On the Origin of Species (John Murray, London, 1859).

[2] D. W. Thompson, On Growth and Form (Cambridge University Press, Cambridge, 1942).

[3] H. R. Hepburn, C. W. W. Pirk, O. Duangphakdee, Honeybee Nests. Composition, Structure Function. (Springer Science& Business Media, Berlin, Heidelberg, 2014).

[4] D. A. Thompson, On Growth and Form (Cambridge University Press, Cambridge, New York, 1945).

[5] T. C. Hales, The honeycomb conjecture. Discrete Comput. Geom. 25, 1–22 (2001).

[6] F. H. Kaatz, A. Bultheel, T. Egami, Order parameters from image analysis: A honeycomb example. Naturwissenschaften 95, 1033 (2008). 18633584 10.1007/s00114-008-0418-4

[7] T. Räz, On the application of the honeycomb conjecture to the bee’s honeycomb. Philos. Math. 21, 351–360 (2013).

[8] H. R. Hepburn, L. A. Whiffler, Construction defects define pattern and method in comb building by honeybees. Apidologie 22, 381–388 (1991).

[9] M.-X. Yang, K. Tan, S. E. Radloff, M. Phiancharoen, H. R. Hepburn, Comb construction in mixed-species colonies of honeybees, Apis cerana and Apis mellifera. J. Exp. Biol. 213, 1659–1664 (2010). 20435816 10.1242/jeb.035626

[10] V. Gallo, L. Chittka, Cognitive aspects of comb-building in the honeybee?. Front. Psychol. 9, 943–949 (2018). 29951014 10.3389/fpsyg.2018.00900 PMC6008556

[11] M. L. Smith, N. Napp, K. H. Petersen, Imperfect comb construction reveals the architectural abilities of honeybees. Proc. Natl. Acad. Sci. 118, e2103605118. (2021). 34312229 10.1073/pnas.2103605118 PMC8346884

[12] L. F. Tóth, What the bees know and what they do not know. Bull. Am. Math. Soc. 70, 468–481 (1964).

[13] C. W. W. Pirk, H. R. Hepburn, S. E. Radloff, J. Tautz, Honeybee combs: Construction through a liquid equilibrium process?. Naturwissenschaften 91, 1–4 (2004). 15257392 10.1007/s00114-004-0539-3

[14] B. L. Karihaloo, K. Zhang, J. Wang, Honeybee combs: How the circular cells transform into rounded hexagons. J. R. Soc. Interface 10, 20130299 (2013). 23864500 10.1098/rsif.2013.0299 PMC3730681

[15] F. Nazzi, The hexagonal shape of the honeycomb cells depends on the construction behavior of bees.. Sci. Rep., 1–6 (2016). 27320492 10.1038/srep28341 PMC4913256

[16] S. S. S. Cardoso , The bee tetragonula builds its comb like a crystal. J. R. Soc. Interface 17, 20200187 (2020). 32693749 10.1098/rsif.2020.0187 PMC7423432

[17] S. Krishna, A. Gopinath, S. M. Bhattacharjee, Ordering and topological defects in social wasps’ nests. Sci. Rep. 12, 1–9 (2022). 35902614 10.1038/s41598-022-16836-6 PMC9334299

[18] D. Jeong, Y. Choi, J. Kim, Modeling and simulation of the hexagonal pattern formation of honeycombs by the immersed boundary method. Commun. Nonlinear Sci. Numer. Simul. 62, 61–77 (2018).

[19] T. Narumi, K. Uemichi, H. Honda, K. Osaki, Self-organization at the first stage of honeycomb construction: Analysis of an attachment-excavation model. Plos One 13, e0205353. (2018). 30356288 10.1371/journal.pone.0205353 PMC6200235

[20] T.-H. Liu, G. Gajewski, C.-W. Pao, C.-C. Chang, Structure, energy, and structural transformations of graphene grain boundaries from atomistic simulations. Carbon 49, 2306–2317 (2011).

[21] S. Kirkpatrick, C. D. Gelatt, M. P. Vecchi, Optimization by simulated annealing. Science 220, 671–680 (1983). 17813860 10.1126/science.220.4598.671

[22] P. J. M. Van Laarhoven, E. H. L. Aarts, Simulated Annealing: Theory and Applications (Springer, pp. 7–15. 1987).

[23] N. Metropolis, A. W. Rosenbluth, M. N. Rosenbluth, A. H. Teller, E. Teller, Equation of state calculations by fast computing machines. J. Chem. Phys. 21, 1087–1092 (1953). 10.1063/5.0309018 41342507

[24] A. Khachaturyan, S. Semenovsovskaya, B. Vainshtein, The thermodynamic approach to the structure analysis of crystals.. Acta Crystallogr. Sec. A: Crystal Phys., Diffraction, Theor. General Crystallogr. 37, 742–754 (1981).

[25] J. Pannetier, J. Bassas-Alsina, J. Rodriguez-Carvajal, V. Caignaert, Prediction of crystal structures from crystal chemistry rules by simulated annealing. Nature 346, 343–345 (1990).

[26] A. T. Brunger, Simulated annealing in crystallography. Annu. Rev. Phys. Chem. 42, 197–223 (1991).

[27] M. D. Rintoul, S. Torquato, Reconstruction of the structure of dispersions. J. Colloid Interface Sci. 186, 467–476 (1997). 9056377 10.1006/jcis.1996.4675

[28] H. Kumar, C. L. Briant, W. A. Curtin, Using microstructure reconstruction to model mechanical behavior in complex microstructures. Mech. Mater. 38, 818–832 (2006).

[29] P. Čapek, V. Hejtmánek, L. Brabec, A. Zikánová, M. Kočiřík, Stochastic reconstruction of particulate media using simulated annealing: Improving pore connectivity. Transp. Porous Media 76, 179–198 (2009).

[30] A. K. Geim, Graphene: Status and prospects. Science 324, 1530–1534 (2009). 19541989 10.1126/science.1158877

[31] K. Kim , Grain boundary mapping in polycrystalline graphene. ACS Nano 5, 2142–2146 (2011). 21280616 10.1021/nn1033423

[32] P. Pieranski, Two-dimensional interfacial colloidal crystals. Phys. Rev. Lett. 45, 569 (1980).

[33] P. Pieranski, L. Strzelecki, B. Pansu, Thin colloidal crystals. Phys. Rev. Lett. 50, 900 (1983).

[34] S. Cai, D. Breid, A. J. Crosby, Z. Suo, J. W. Hutchinson, Periodic patterns and energy states of buckled films on compliant substrates. J. Mech. Phys. Solids 59, 1094–1114 (2011).

[35] M. Brojan, D. Terwagne, R. Lagrange, P. M. Reis, Wrinkling crystallography on spherical surfaces. Proc. Natl. Acad. Sci. U.S.A. 112, 14–19 (2015). 25535355 10.1073/pnas.1411559112 PMC4291636

[36] T. Hahn, U. Shmueli, J. C. W. Arthur, International Tables for Crystallography (Reidel Dordrecht, Dordrecht, 1983), vol. 1.

[37] D. G. Brandon, The structure of high-angle grain boundaries. Acta Metall. 14, 1479–1484 (1966).

[38] H. Van Swygenhoven, Grain boundaries and dislocations. Science 296, 66–67 (2002). 11935012 10.1126/science.1071040

[39] O. V. Yazyev, S. G. Louie, Topological defects in graphene: Dislocations and grain boundaries. Phys. Rev. B. 81, 195420. (2010).

[40] T. O. E. Skinner, D. G. A. L. Aarts, R. P. A. Dullens, Grain-boundary fluctuations in two-dimensional colloidal crystals. Phys. Rev. Lett. 105, 168301. (2010). 21231020 10.1103/PhysRevLett.105.168301

[41] P. Y. Huang , Grains and grain boundaries in single-layer graphene atomic patchwork quilts. Nature 469, 389–392 (2011). 21209615 10.1038/nature09718

[42] S. Gokhale, K. H. Nagamanasa, R. Ganapathy, A. K. Sood, Grain growth and grain boundary dynamics in colloidal polycrystals. Soft. Matter. 9, 6634–6644 (2013).

[43] T. D. Edwards, Y. Yang, D. J. Beltran-Villegas, M. A. Bevan, Colloidal crystal grain boundary formation and motion. Sci. Rep. 4, 1–8 (2014). 10.1038/srep06132 PMC4138518 25139760

[44] C. Ophus, A. Shekhawat, H. Rasool, A. Zettl, Large-scale experimental and theoretical study of graphene grain boundary structures. Phys. Rev. B. 92, 205402. (2015).

[45] M. Bowick, A. Cacciuto, D. R. Nelson, A. Travesset, Crystalline order on a sphere and the generalized Thomson problem. Phys. Rev. Lett. 89, 185502. (2002). 12398614 10.1103/PhysRevLett.89.185502

[46] F. L. Jiménez, N. Stoop, R. Lagrange, J. Dunkel, P. M. Reis, Curvature-controlled defect localization in elastic surface crystals. Phys. Rev. Lett. 116, 104301. (2016). 27015484 10.1103/PhysRevLett.116.104301

